# Improving adolescents’ dietary behavior through teacher-delivered cancer prevention education: a school-based cluster randomized intervention trial in urban Rajasthan

**DOI:** 10.1186/s12889-024-18114-8

**Published:** 2024-02-28

**Authors:** Ankit Mittal, Neeti Rustagi, Prasanna Thirunavukkarasu, Santu Ghosh, Pankaja Raghav

**Affiliations:** 1 Department of Community Medicine and Family Medicine, All India Institute of Medical Sciences, Jodhpur, Rajasthan India; 2 Department of Biostatistics, St Johns Medical College, Koramangala, Bangalore, India

**Keywords:** School based intervention, Theory of planned behavior, Nutrition education, Processed food, Prospective study, Diet and cancer

## Abstract

**Background:**

Dietary practices are one of the most common modifiable risk factors for cancers. Foods rich in dietary fibers are considered protective, meanwhile fast & junk foods are risk for common cancers. Adolescence period is marked by habit formation and is thus suited for delivering behavioral intervention. Schools offer an optimal setting for planning and executing these interventions to a large number of adolescents.

**Objective:**

To assess the effectiveness of a teacher-delivered cancer-prevention education in changing dietary behaviors of school going adolescents.

**Methods:**

A cluster randomized trial was conducted in government secondary and senior secondary schools with schools as clusters. A minimum required sample of 1032 students was estimated from 16 schools with 1:1 allocation in intervention and non-intervention groups. Dietary behaviors were recorded as dichotomous variable. The determinants were recorded as per theory of planned behavior framework using Likert-scale. Two teachers from each intervention school were trained to deliver cancer prevention education with focus on role of dietary behavior. Pre-post training assessment of teachers’ knowledge towards common cancers was done using a self-administered questionnaire. Gender adjusted difference-in-difference analysis was done to assess intervention effect on both healthy and unhealthy behaviors.

**Results:**

In selected schools all students from classes 8 to 10 were approached and a total of 1224 students were enrolled, of whom 1096 completed the study. The study recorded significant improvement in scores of students from intervention group compared to non-intervention group for their attitude, subjective norms, perceived behavioral control and intention towards consuming healthy and avoiding unhealthy foods. The intervention was effective in significantly improving the proportion of students limiting fried/fast/packed food & sugar sweetened beverages (OR:1.51, 95%CI:1.08,2.12,p:0.017), and consuming fruits & vegetables daily (OR:1.55, 95%CI:1.08,2.22, p:0.017) while adjusting effect of gender.

**Conclusion:**

Classroom-based cancer prevention education delivered through teachers during regular working hours is effective in improving dietary behaviors and its determinants among adolescent students. Thus, we recommend integrating a section focusing on the role of diet in cancer prevention and other lifestyle diseases in the existing school curriculum.

**Trial Registration:**

The trial was registered under Clinical Trial Registry-India with registration number CTRI/2018/12/016586, dated-10/12/2018.

**Supplementary Information:**

The online version contains supplementary material available at 10.1186/s12889-024-18114-8.

## Introduction

Prevention is considered as the most effective strategy to mitigate the problem of cancer. More than half of all cancer deaths can be attributed to risky behavioral choices [1]. A report by American Association for Cancer Research (AACR) has attributed approximately one fourth of cancers to unhealthy dietary practices and its consequences like obesity [2]. Fiolet et al. found an association between ultra-processed food in diet and higher overall cancer risk with a hazard ratio of 1.12 for a 10% increment in the proportion of ultra-processed food in the diet [3]. For more specific cancers, poor food choice is directly associated with gastrointestinal cancers, and through childhood obesity with breast cancers [4, 5]. Fast food consumption is associated with increased risk of noncommunicable disease [6]. The National Non-communicable Disease Monitoring Survey (NNMS) 2017–2018, India reported high prevalence of consuming fried foods (52.9%, 95%CI: 46.1%, 59.6%), chips (58.3%, 95%CI: 52.4%, 63.9%), cold aerated drinks (23.2%, 95%CI: 18.3%, 28.9%) and energy drinks (11.6%, 95%CI: 7.4%, 17.7%) among urban adolescents [7]. Dietary fibers, the main source of which are fruits and vegetables are considered beneficial in preventing several noncommunicable diseases including cancers [8, 9]. The consumption of fruits by adolescents (10 to 19 years) was reported at only 59.1% in urban India by the Comprehensive National Nutrition Survey (CNNS) 2016–2018 [10]. These nationwide surveys indicate the high magnitude of unhealthy dietary behavior among Indian adolescents.

According to World Health Organization (WHO), adolescence is an age of establishing patterns of behaviors including dietary habits [11]. During this phase, adolescents develop knowledge and skills, form own ideas and habits, and is considered as a perfect time to deliver the intervention to them [12, 13]. Schools serve as potential target to plan and implement behavior change communication strategies as they have the capability and the necessary tools to provide a positive impact on students’ health [14]. Teachers have played role of key players for bringing cognitive and behavioral changes aiming to promote healthy lifestyle in the society and have proven effective [15, 16]. The engagement and training of teachers in delivering health promotion education to students might prove beneficial in sustaining the behavior change towards dietary practices over long duration. This is relatively an unexplored area of research in Indian setting. Thus, the current study was done with objective to assess the effectiveness of a teacher-delivered cancer-prevention education in changing dietary behaviors of school going adolescents.

## Materials and methods

### Study design and setting

A cluster randomized intervention trial was conducted from February 2019 to January 2020 among students of government secondary and senior secondary schools of urban Jodhpur with schools as clusters. Jodhpur city is district headquarter, centrally situated in the western region of Rajasthan state, and comes under the arid zone of state. The city has a population above 1 million and an area of about 233 km^2^ [17]. As of January 2019, the city had a total of 68 government schools having classes from 8th to 10th. Permissions were obtained from the office of District Education Officer (DEO)-secondary education and individual school authorities.

### Sample size

Shah et al. reported post intervention change in dietary behavior of adolescent students ranging from 8 to 22% for consuming healthy and limiting unhealthy food [18]. Thus, for current study, a near mean value of 15% was assumed as expected change in dietary behavior. Comprehensive national nutrition survey (2016–2018) by the Ministry of Health and Family Welfare, India, reported consumption of fruits among adolescents to be 40% [10]. Thus for sample size calculation, baseline consumption of 40% with expected change of at least 15% to be achieved in the intervention group at 80% power and two-sided significance of 95% was considered. Considering the above assumptions, the required sample size without clustering was calculated to be 338 (169 in each arm).

The average cluster size of 59 students was considered for sample size calculation in urban secondary and senior secondary schools based on recommendation of DEO-secondary education. The value of intra cluster correlation coefficient (ICC) for human studies is assumed as 0.01 to 0.02 and small value of ICC implies that within cluster variance is greater than between cluster variance [19]. A slightly higher ICC of 0.03 was considered for current study as according to ASER 2019 government school students are generally from lower socioeconomic background and have less educated parents, thus form a homogenous group [20]. 

The design effect was calculated [*DE = 1 + p(n-1); p* is ICC and *n* is average cluster size] as 2.745 which was multiplied to effective sample size of 338 students and resulted in 928 (464 in each group) students [21, 22]. Considering 10% non-response, a sample size of 1032 was estimated with 516 participants each in intervention and non-intervention arm.

### Sampling design

For cluster sampling, a sampling frame of 68-government secondary and senior secondary schools of Jodhpur city was prepared. For randomization, schools were arranged in increasing order of their unique ‘Unified District Information System for Education’ (UDISE) code provided to each school by the Ministry of Education, rather than their names or number of students to prevent selection bias. Using “=RAND()” function of MS Excel, random numbers were assigned to these schools and were rearranged in ascending order of these random numbers to select 16 schools. All 16 school authorities agreed to participate in the study and were randomly allocated to intervention and non-intervention groups with 1:1 allocation ratio. As result of random allocation, each group had 2 secondary schools and 6 senior secondary schools. The students from classes 8 to 10 were approached to ensure reaching out to adolescents of age 13 to 17 years [23]. All students from these classes were enrolled in the study. Enrolment of two teachers per intervention school was recommended by school authorities (principal, vice-principal, head teacher) based on feasibility assessed in integrating the module within regular school curriculum of selected classes. Enrolled teachers were trained to deliver cancer prevention education to students of selected classes.

### Data collection tools

**Fig. 1:**
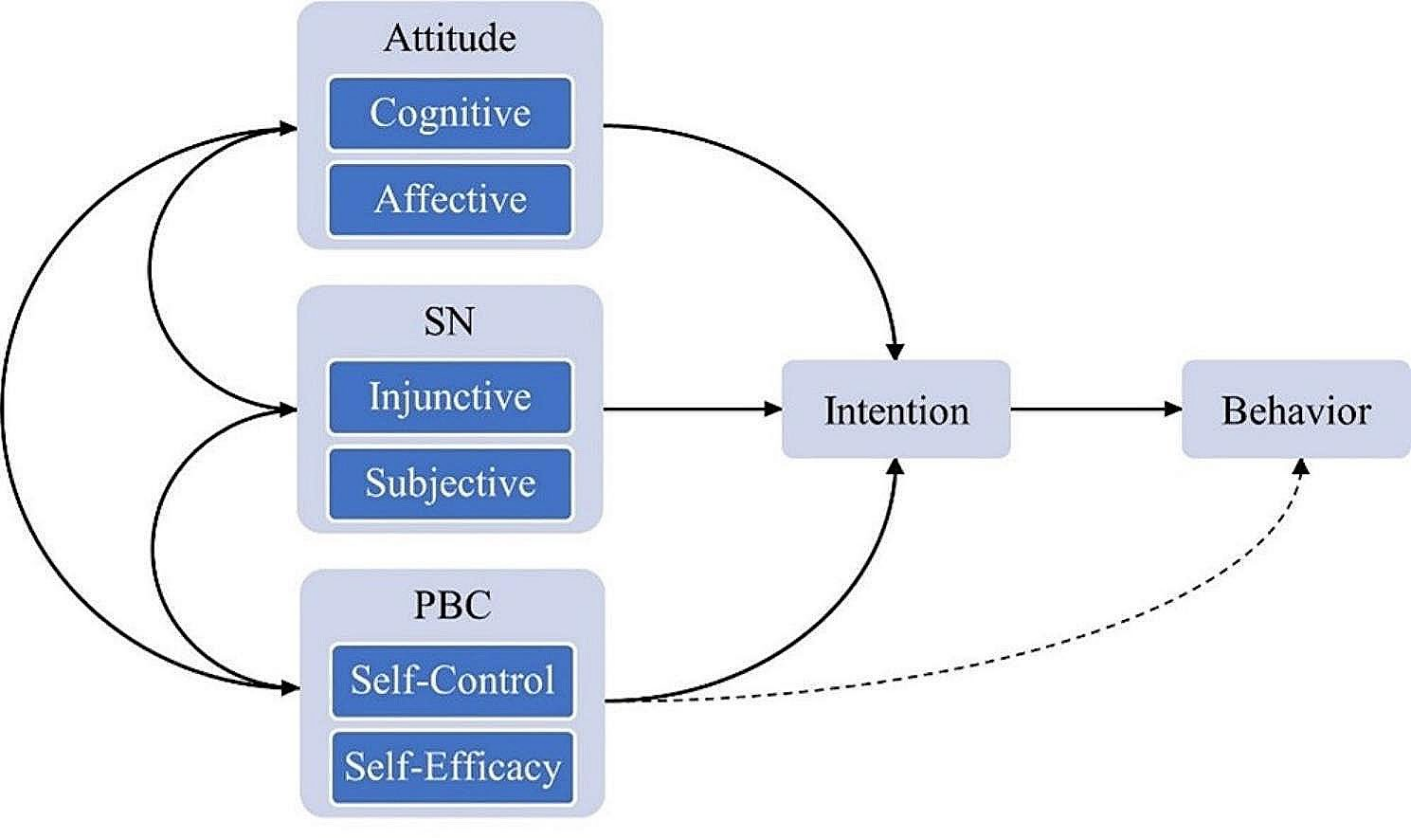
Framework of theory of planned behavior

The theoretical framework of Theory of Planned Behavior (TPB) was used to assess adolescents’ dietary behavior and its determinants. TPB predicts behavior by assessing participant’s attitude, subjective norms (SN), perceived behavioral control (PBC), and intention towards specific behavior (Fig. 1) [24]. The Questionnaire for students had 3 parts – (1) sociodemographic details, (2) TPB constructs assessing consumption of fruits & vegetables, and (3) TPB constructs assessing limiting consumption of fried/fast/packed food & sugar sweetened beverages (SSB). (Annexure 1)

Items in the form of 5-point Likert scale were framed to assess Attitude, SN, and PBC among students with 2 items for each construct. The Likert scale was scored from 1 to 5 with 3 being the point of neutral response, 4 and 5 indicating positive, and less than 3 being negative response. Thus, summative scores for each construct were on a scale of 2 to 10 where a score of 8 and above as *Good*, while a score of 2 to 7 as *Poor* were used as cut off for composite scores. For attitude, one item each assessed cognitive and affective component [Cognitive - “What effect the *target behavior* will have on health”; Affective - “How would you like to adopt the *target behavior*”] was used. For SN, one item each to assess injunctive norms [“My friends and family members support *target behavior*”] and descriptive norms [“Most healthy people practice *target behavior*”] was used. To assess the PBC, one item each was included for self-control [“Adopting *target behavior* depends on me”] and self-efficacy [“I am confident in adopting *target behavior*”]. The intention towards dietary behaviors was assessed by a single item [“I want to adopt the *target behavior*”] scaled on a 5-point Likert scale with scores of 4 and more indicating a positive response towards desired outcomes, hence interpreted as *Good*, while 1 to 3 as *Poor*. Behavioral outcomes of the students were assessed in the form of dichotomous True/False questions and proportions were calculated. The questionnaire was developed in the local language and response process validity was assessed to ensure clarity and comprehension of questions by students [25]. 

Knowledge of teachers was assessed through a questionnaire comprising of multiple-choice questions, match the columns and true or false questions with total score of 27 marks. The following areas were assessed - biology of cancers, risk factors and danger signs, palliative care, and diet and health both pre and post training using a self-administered questionnaire. (Annexure 1)

### Intervention

**Fig. 2:**
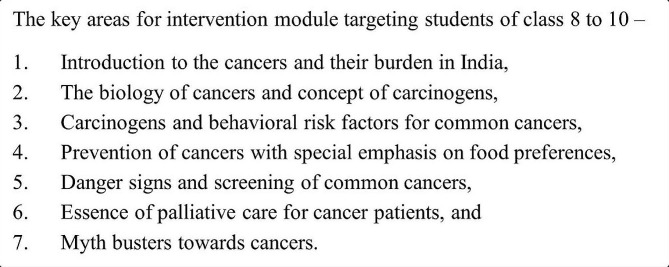
Key areas of intervention module

The intervention module was developed through multiple rounds of consultation with physicians specialized in community medicine or public health with expertise in fields of cancer prevention, diet & nutrition, and adolescent health. Experts were reached out through e-mail for the inputs regarding the module on receiving their consent to review. The whole module, including specific sessions to be imparted by the teachers to students in classroom settings was shared. All the inputs by experts were incorporated. A total of 7 key areas were identified for developing the intervention module (Fig. 2). Intervention was designed in the form of stories, case studies, graphical illustrations, and examples from day-to-day life to effectively engage students.

For the intervention group, training of nominated teachers was done in each school in a session lasting for 60 to 90 min within the working hours of schools. Through pre and post-test, the understanding of teachers about all selected topics was evaluated. These trained teachers subsequently delivered the components of the module to selected classes during regular working hours and as part of the existing school curriculum. No extra hours were utilized to implement the intervention. No such training was provided in schools from non-intervention group and regular school curriculum was followed in these schools.

### Data collection

All the students were contacted twice for data collection 6 months apart. In intervention group schools, a gap of 6 months after training of teachers was ensured for endline data. Before administering the questionnaire, investigator explained that the student’s participation was voluntary and to be considered non evaluating as the scores were not used for any academic evaluation. The presence of investigator during data collection ensured easy understanding and addressing of any doubts raised by students.

### Outcome variables

Outcome variables of the study were to evaluate effectiveness of intervention in form of change in proportion of students consuming fruits & vegetables daily, change in proportion of students limiting consumption of fried/fast/packed food & sugar sweetened beverages to maximum twice a week, change in scores of constructs of TPB towards these behavior among students, and change in knowledge of teachers towards key intervention areas regarding cancers.

### Statistical considerations

Data was entered and analyzed using Microsoft Excel 2016 and Statistical Package for Social Sciences (SPSS) version 23. For baseline comparison of intervention and non-intervention group, independent t test was used for constructs of TPB. The changes in scores of TPB constructs from baseline to endline were analyzed with paired t test, and the comparison of score change between groups was done using difference of differences analysis with independent t test. For evaluation of intervention effect on behavior, difference in differences (DID) analysis was used to calculate odds ratio with behavior as dependent variable, and group, time, and interaction of group and time as independent variables. All statistical significance was tested at 5% level of significance, and 95% confidence limits were provided for mean difference and odds ratio.

**Fig. 3:**
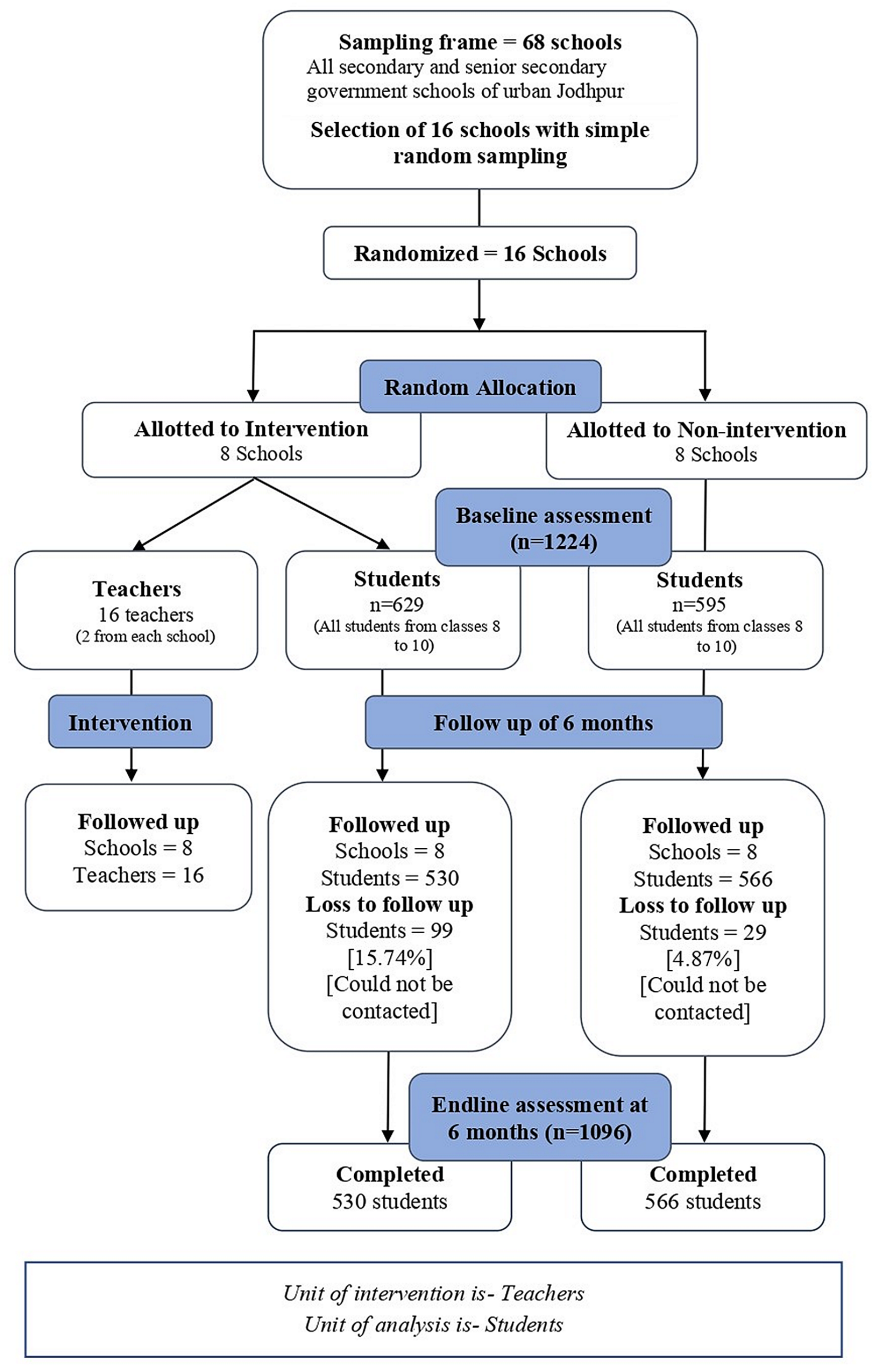
Consort flow diagram of participants

## Results

From 16 schools, a total of 1224 students were enrolled, of whom 1096 completed the study.(Fig. 3) The baseline sociodemographic characteristics of these students were found to be comparable.(Table 1) At the endline, the average cluster size in the intervention and non-intervention group were 66.25 and 70.75 students respectively yielding an average cluster size of 68.5 students for selected schools.

The majority of students belonged to the age group of 14 to 16 years, with a mean age of 15.23 ± 1.14 years in the intervention group and 15.35 ± 1.17 years in the non-intervention group. Students in the selected classes were predominantly girls. There was no significant difference in both the groups in the three grades included.

A total of 16 teachers from 8 intervention schools were enrolled and trained for delivery of modular teaching. The age of teachers ranged from 26 to 46 years with a mean age of 35.13 ± 5.79 years. There were 11 (68.8%) female teachers, and 4 (25.0%) were science graduates. The training in intervention module significantly improved the scores of teachers for knowledge in all components of the questionnaire at post-test. (Annexure 2)

**Table 1:** Sociodemographic characteristics of participants who completed the study (*n* = 1096)

Variable	*Intervention group (n = 530)*	*Non-intervention group (n = 566)*	*P* value^a^
Age in years (Mean ± SD)	15.23 ± 1.14	15.35 ± 1.17	0.095
***Age category***, *n (%)*			
≤ 13 years	52 (9.8%)	52 (9.2%)	0.085
14 to 16 years	423 (79.8%)	430 (76.0%)	
≥ 17 years	55 (10.4%)	84 (14.8%)	
***Gender***, *n (%)*			
Male	148 (27.9%)	153 (27.0%)	0.741
Female	382 (72.1%)	413 (73.0%)	
***Distribution of participants by class***, *n (%)*			
8th std	91 (17.2%)	85 (15.0%)	0.217
9th std	269 (50.8%)	272 (48.1%)	
10th std	170 (32.1%)	209 (36.9%)	
***Education of father***^**b**^, *n (%)*			
Illiterate	79 (14.9%)	78 (13.8%)	0.475
Primary school	99 (18.7%)	112 (19.8%)	
Middle school	101 (19.1%)	117 (20.7%)	
Secondary school	148 (27.9%)	135 (23.9%)	
Senior secondary and above	96 (18.1%)	83 (14.7%)	
Do not know/Did not respond	7 (1.3%)	41 (7.2%)	
***Education of mother***^**b**^, *n (%)*			
Illiterate	219 (41.3%)	179 (31.6%)	0.05
Primary school	119 (22.5%)	147 (26.0%)	
Middle school	94 (17.7%)	114 (20.1%)	
Secondary school	66 (12.5%)	58 (10.2%)	
Senior secondary and above	27 (5.1%)	25 (4.4%)	
Do not know/Did not respond	5 (0.9%)	43 (7.6%)	
***Father’s occupation***^**b**^, *n (%)*			
Unskilled/Semiskilled	191 (36.0%)	193 (34.1%)	0.735
Skilled	230 (43.4%)	224 (39.6%)	
Clerical/semi-professional/ Professional	100 (18.9%)	111 (19.6%)	
Do not know/Did not respond	9 (1.7%)	38 (6.7%)	
***Mother’s occupation***^**b**^, *n (%)*			
Unemployed/Housewife	438 (82.6%)	459 (81.1%)	0.53
Unskilled/Semiskilled	31 (5.8%)	25 (4.4%)	
Skilled	46 (8.7%)	41 (7.2%)	
Clerical/Semi-professional/ Professional	10 (1.9%)	15 (2.7%)	
Do not know/Did not respond	5 (0.9%)	26 (4.6%)	
***Monthly family income in INR***, *n(%)*			
Up to 10,000	219 (41.3)	200 (35.3)	NA^c^
10,001 to 20,000	133 (25.1)	64 (11.3)	
More than 20,000	58 (10.9)	35 (6.2)	
Do not know/Did not respond	120 (22.6)	267 (47.2)	

^***a***^ *Chi-square test is used for age category, gender, distribution of participants by class, education and occupation of father and mother. Independent student t test is used for mean age. Do not know/Did not respond is not included in analysis.*

^***b***^ *Mother and Father’s education and occupation is as provided by students*

^***c***^ *Not analyzed due to large missing data*

### Dietary behaviors and its determinants on TPB

At the baseline the non-intervention group had significantly higher score for attitude towards consuming fruits & vegetables daily (IG: 8.88 ± 1.17, NIG: 9.16 ± 1.13), whereas the score of limiting consumption of fried/fast/packed food & SSB was better for those in intervention group (IG: 6.40 ± 2.25, NIG: 5.83 ± 2.45). The intervention group also demonstrated a higher score for SN towards limiting unhealthy foods (IG: 7.40 ± 1.81, NIG: 7.05 ± 2.02). All other constructs of TPB were comparable at baseline for both the groups. (Annexure 3) At the endline the difference of difference analysis revealed that the change in scores for TPB constructs in intervention group were significantly higher than the change in non-intervention schools. (Table 2)

At the baseline, less than half (529 of 1096, 48.3%) of the participants reported to limit consumption of unhealthy foods and were comparable for both intervention and non-intervention groups (IG:49.1%, NIG:47.5%). (Annexure 4) At the endline the intervention group reported limiting unhealthy foods by 58.7% students, compared to 47.0% in the non-intervention group.

At baseline a total of 61.2% (671 of 1096) participants reported having both fruits & vegetables daily and proportion of participants was comparable for both the groups (IG:59.6%, NIG:62.7%). (Annexure 4) The intervention was able to increase the proportion of students to 74.0% in intervention group compared to 67.7% in non-intervention group.

**Table 2:** Effect of intervention on scores for TPB constructs towards dietary behaviors

TPB Measures	*Intervention group (n = 530)*			*Non-Intervention group (n = 566)*			Difference of differences^b^	
	Baseline Mean (SD)	Endline Mean (SD)	Mean difference^a^ (95% CI)	Baseline Mean (SD)	Endline Mean (SD)	Mean difference^a^ (95% CI)	Mean (95% CI)	*P* value
***Limiting consumption of fried/fast/packed foods & sugar sweetened beverages***								
Attitude	6.40 (2.25)	7.69 (2.11)	1.29 (1.12, 1.46)*	5.83 (2.45)	6.16 (2.32)	0.33 (0.24, 0.44)*	0.95 (0.75, 1.15)	< 0.001*
SN	7.40 (1.81)	8.11 (1.73)	0.70 (0.59, 0.82)*	7.05 (2.02)	7.19 (1.87)	0.14 (0.05, 0.23)*	0.56 (0.42, 0.71)	< 0.001*
PBC	8.45 (1.80)	8.89 (1.53)	0.45 (0.34, 0.55)*	8.46 (1.96)	8.35 (1.93)	-0.11 (-0.22, -0.01)*	0.56 (0.41, 0.71)	< 0.001*
Intention to limit consumption	4.05 (1.25)	4.36 (0.98)	0.31 (0.23, 0.38)*	4.06 (1.36)	4.11 (1.19)	0.05 (-0.02, 0.11)	0.26 (0.16, 0.36)	< 0.001*
***Consuming fruits & vegetables daily***								
Attitude	8.88 (1.17)	9.12 (1.01)	0.24 (0.18, 0.31)*	9.16 (1.13)	9.18 (1.06)	0.02 (-0.03, 0.07)	0.23, 0.04 (0.15, 0.31)	< 0.001*
SN	8.67 (1.55)	9.04 (1.28)	0.36 (0.28, 0.44)*	8.67 (1.57)	8.66 (1.55)	0.00 (-0.06, 0.06)	0.37, 0.05 (0.27, 0.47)	< 0.001*
PBC	8.81 (1.62)	9.24 (1.21)	0.43 (0.34, 0.52)*	8.82 (1.64)	8.81 (1.64)	-0.01 (-0.07, 0.06)	0.44, 0.06 (0.32, 0.55)	< 0.001*
Intention to consume daily	4.52 (0.91)	4.68 (0.69)	0.16 (0.11, 0.21)*	4.53 (0.96)	4.55 (0.89)	0.01 (-0.03, 0.06)	0.15, 0.04 (0.07, 0.22)	< 0.001*

*SN: Subjective Norms, PBC: Perceived Behavioral Control*

* *Statistically significant at confidence level of 95%*

^***a***^ *Mean difference and 95% CI from baseline to endline using paired t-test*

^*b*^ *Difference of mean differences for intervention and non-intervention groups using independent t-test*

### Difference in differences analysis

Odds of consuming both fruits & vegetables daily and limiting fried/fast/packed food & SSB was significant in intervention group as per DID analysis. The final model when adjusted for gender to account for preponderance of girl students in the study, revealed significant improvement of dietary behaviors in intervention group for both limiting fried/fast/packed food & SSB (OR:1.51, 95%CI:1.08,2.12, p:0.017) and daily consumption of fruits & vegetables (OR:1.55, 95%CI:1.08,2.22, p:0.017). (Table 3)

**Table 3:** Effect of intervention on dietary behaviors of students using difference in differences analysis

Behavioral outcomes	OR (95% CI)	*P* value	aOR (95% CI)^a^	*P* value
Limiting fried/fast/packed food & sugar sweetened beverages	1.72 (1.19, 2.50)	0.004*	1.51 (1.08, 2.12)	0.017*
Limiting at least one of fried/fast food and packed food & sugar sweetened beverages	1.33 (0.86, 2.05)	0.203	1.15 (0.78, 1.71)	0.474
Consuming fruits & vegetables daily	1.65 (1.10, 1.47)	0.015*	1.55 (1.08, 2.22)	0.017*
Consuming at least one of fruits & vegetables daily	1.22 (0.61, 2.42)	0.579	1.22 (0.64, 2.31)	0.544

*-Difference in differences (DID) analysis is done with behavior as dependent variable (1-Desired behavior, 0-Not desired behavior) and group (1- Intervention group; 0- Non-intervention group), time (1-Endline, 0-Baseline), and interaction of group and time as independent variables.*

^*a*^ *DID model adjusted for gender*

* Statistically significant at 95% CL

### Barriers and facilitators of intervention delivery

Barriers and facilitators expressed by teachers in implementing modules are discussed in Table 4.

**Table 4:** Barriers and facilitators of intervention delivery

Facilitators	- Nomination of teachers by school authorities ensured availability of required resources and time to implement the intervention as part of regular curriculum. - Easy language of module was appreciated, and teachers felt confident in implementing module even without prior training. - Pictorial representation and examples from day-to-day life was reported to be helpful in explaining cancer and risk factors to students
Barriers	- Intervention module not being part of academic evaluation limits the efforts and attention of students. - Reaching out to parents / family of students for behavior change is essential and must be integrated. - Perceived burden by teachers due to nonacademic responsibilities (surveys, election duties etc.) - Engaging two teachers for implementing intervention was considered insufficient and need of more number of teachers to facilitate discussion related to intervention implementation was expressed.

## Discussion

Focusing on adolescent knowledge and formation of preference for nutritive foods is critical as it is likely to culminate as healthy preferences in adulthood [18]. Developing and implementing nutritional behavioral intervention program for resource-constrained schools is found to be challenging yet feasible for influencing students’ food choices [18, 26]. Effectiveness of engaging in-service trained teachers as interventionists for influencing students’ dietary behaviors is an under-researched area especially in schools catering to students of dis-advantaged background [26–28]. Effective implementation of planned nutritional intervention requires engagement of teachers through school authorities to facilitate availability of required resources and support during regular school hours [27, 29]. 

For the current study, a total of 530 and 566 students participated in intervention and non-intervention groups while the required effective sample size was 464 in each group. The sociodemographic characteristics for both groups at end of study were found to be comparable and loss to follow up of 10.5% (15.7% in IG and 4.9% in NIG) was observed at end line assessment. As reported by Carter et al., differential participation in longitudinal studies has minimal effect on exposure-outcome association, thus differential loss of participants in our study is unlikely to cause any major implications on behavior change observed [30]. 

Poor attitude, perceived control and favorable subjective norms toward consumption of fried/fast/packed food & SSB highlight low awareness regarding health hazards of junk foods and it’s acceptance among adolescents [31]. The current study supports the utility of theoretical framework of TPB for targeting adolescent dietary behaviors in Indian setting; all TPB constructs (attitude, SN, PBC and intention) significantly improved in our study as was also reported for studies engaging students of diverse age groups [32, 33]. 

The current study established effectiveness of engaging teachers for delivering healthy diet intervention for improved behavior changes among students of under-resourced and disadvantaged background for duration of at least 6 months [28]. These results are comparable to investigator delivered nutritional educational intervention among school students [34, 35]. Considering less resource consuming, minimally controlled and self-sustaining delivery of nutritional intervention method, the results of current study provide evidence towards incorporating nutrition–education interventions through teachers within regular school curriculum [26]. The engagement of teachers in delivering health promotion intervention and its reinforcement as part of regular curriculum may prove effective towards influencing and sustaining healthy dietary behaviors over prolonged duration in adolescents. Further research assessing the combined role of peers, teachers, and parents in influencing adolescent dietary behavior to address lifestyle disease risk factors is thus needed and cohort studies in diverse school settings are warranted to establish evidence in this regard.

Predominantly girl students participated in both intervention (72.1%) and non-intervention (73.0%) schools. Higher enrolment of girls in government schools is similarly observed in a nationwide survey of ASER 2019 [20]. At endline, the intervention group reported significant improvement in proportion of students consuming both fruits & vegetables daily and limiting consumption of both groups of unhealthy foods as reported by other intervention studies among school students [36, 37]. Females are reported to have higher motivation towards healthy diet as compared to male students [38]. The current study revealed that improvement in dietary behavior was significant among intervention schools even after adjusting for gender of students. Limited literature is available regarding gender influence on adoption of healthy behavior. Thus, conducting similar studies with focus on gender disaggregated data in schools is needed for framing gender specific recommendations among students.

Our study is one of the few attempts to apply a theoretical approach in the form of TPB to explain adolescent dietary behavior and the role of teachers as effective agents of behavior change. One of the key limitations of our study is the use of a self-administered questionnaire. The influence of social desirability bias was reduced by measures such as providing a friendly environment during data collection, explaining to students the non-evaluative nature of the questionnaire, and ensuring the anonymity of participants. Further, it is unlikely to influence the estimation of intervention effect, since it would have been same for both the groups [27]. 

Based on findings of the current study, we recommend adapting school-curriculum and engaging school authorities and teachers for implementing nutritional interventions for healthy dietary behavior towards prevention of lifestyle diseases including cancers.

## Conclusion

The study establishes the effectiveness of a teacher delivered cancer prevention - nutrition based intervention in increasing proportion of students adopting healthy dietary behavior and influencing determinants of dietary behavior assessed as per TPB framework in resource constrained schools. Thus, integrating a section focusing on the role of diet in cancer prevention and other lifestyle diseases in the existing school curriculum through in-service trained teachers is recommended.

### Electronic supplementary material

Below is the link to the electronic supplementary material.

Supplementary Material 1

Supplementary Material 2

Supplementary Material 3

Supplementary Material 4

## Data Availability

The datasets used and/or analyzed during the current study are available from the corresponding author on reasonable request.
