# Supplementary material for: Improving adolescents’ dietary behavior through teacher-delivered cancer prevention education: a school-based cluster randomized intervention trial in urban Rajasthan
Source: BMC Public Health. 2024 Feb 28;24:630. doi: 10.1186/s12889-024-18114-8 (PMC10900637; doi:10.1186/s12889-024-18114-8)
Supplement: Supplementary file 1 — Supplementary Material 1 [file 12889_2024_18114_MOESM1_ESM.docx]

1. **Questionnaire for Students’ Dietary Practices**

**Answer all questions and write your response in box provided:**

| **a** | **Details of student:** | | |
| --- | --- | --- | --- |
| 1 | Name |  | |
| 2 | Age |  | |
| 3 | Gender | Male | Female |
| 4 | Class |  | |
| 5 | Educational qualification of father |  | |
| 6 | Occupation of father |  | |
| 7 | Educational qualification of mother |  | |
| 8 | Occupation of mother |  | |
| 9 | Average monthly income of family |  | |

| **b** | **If fruits and vegetables are consumed at least once daily:** | **Response** |
| --- | --- | --- |
| 1 | **How will it be for health?**  Very good          Good           Can’t say                Bad            Very Bad   1. (2) (3) (4) (5) |  |
| 2 | **How would you like it?**  Very good          Good           Can’t say                Bad            Very Bad   1. (2) (3) (4) (5) |  |
| 3 | **My friends and family members will support this.**  Completely agree Somewhat agree Can’t say Somewhat Disagree Completely disagree  (1) (2) (3) (4) (5) |  |
| 4 | **Most healthy people consume fruits and vegetables at least once per day.**  Completely agree Somewhat agree Can’t say Somewhat Disagree Completely disagree  (1) (2) (3) (4) (5) |  |
| 5 | **I want to make this change in my diet.**  Completely agree Somewhat agree Can’t say Somewhat Disagree Completely disagree  (1) (2) (3) (4) (5) |  |
| 6 | **I am confident that I can make this change in my diet.**  Completely agree Somewhat agree Can’t say Somewhat Disagree Completely disagree  (1) (2) (3) (4) (5) |  |
| 7 | **Making this change in my diet depends on me.**  Completely agree Somewhat agree Can’t say Somewhat Disagree Completely disagree  (1) (2) (3) (4) (5) |  |
| 8 | **In past one week I have consumed fruits at least once per day.**  True False  (1) (2) |  |
| 9 | **In past one week I have consumed vegetables at least once per day.**  True False  (1) (2) |  |

| **c** | **If consumption of fried foods, fast foods, packed foods and sugar sweetened beverages is limited to maximum twice a week:** | **Response** |
| --- | --- | --- |
| 1 | **How will it be for health?**  Very good          Good           Can’t say                Bad            Very Bad   1. (2) (3) (4) (5) |  |
| 2 | **How would you like it?**  Very good          Good           Can’t say                Bad            Very Bad   1. (2) (3) (4) (5) |  |
| 3 | **My friends and family members will support this.**  Completely agree Somewhat agree Can’t say Somewhat Disagree Completely disagree  (1) (2) (3) (4) (5) |  |
| 4 | **Most healthy people limit consumption of these food groups to maximum twice a week.**  Completely agree Somewhat agree Can’t say Somewhat Disagree Completely disagree  (1) (2) (3) (4) (5) |  |
| 5 | **I want to make this change in my diet.**  Completely agree Somewhat agree Can’t say Somewhat Disagree Completely disagree  (1) (2) (3) (4) (5) |  |
| 6 | **I am confident that I can make this change in my diet.**  Completely agree Somewhat agree Can’t say Somewhat Disagree Completely disagree  (1) (2) (3) (4) (5) |  |
| 7 | **Making this change in my diet depends on me.**  Completely agree Somewhat agree Can’t say Somewhat Disagree Completely disagree  (1) (2) (3) (4) (5) |  |
| 8 | **In past one week I have limited consumption of fried foods and fast foods to maximum twice a week.**  True False  (1) (2) |  |
| 9 | **In past one week I have limited consumption of packed foods and sugar sweetened beverages to maximum twice a week.**  True False  (1) (2) |  |

1. **Questionnaire for Teachers**

**Mark the single correct option for following questions (Q1-Q7):**

1. **Cancer is a dangerous disease, which can be defined as- (1 Mark)**
   1. Controlled division of cells (controlled increase in number of cells)
   2. Uncontrolled division of cells (uncontrolled increase in number of cells)
   3. Uncontrolled growth of a cell (uncontrolled increase in size of cells)
   4. Controlled growth of a cell (controlled increase in size of cells)
2. **Which of the following statements is correct? (1 Mark)**
   1. Malignant cancer can spread to distant places in the body through blood.
   2. Benign cancer tends to spread from its origin to distant organs.
   3. Metastatic cancers are which stay confined to the place of origin.
   4. Benign cancers are more dangerous than metastatic cancers as they can spread to other organs also.
3. **Deepika is an 11th class student. Her height is 158cm. What should be his approximate ideal body weight for her? (1 Mark)**
   1. 48 kg
   2. 58 kg
   3. 68 kg
   4. 38 kg
4. **Match the following – (1 Mark)**

| **Colum A** | **Colum B** |
| --- | --- |
| 1. Tobacco chewing | i. Gastric cancer |
| 1. Spicy food | ii. Lung cancer |
| 1. Family history | iii. Oral Cancer |
| 1. Smoking | iv. Breast cancer |

- 1. A-iv, B-ii, C-i, D-iii
  2. A-i, B-iii, C-ii, D-iv
  3. A-ii, B-iv, C-iii, D-i
  4. A-iii, B-i, C-iv, D-ii

1. **Match the following for a balanced diet – (1 Mark)**

| **Colum A** | **Colum B** (Daily requirement) |
| --- | --- |
| 1. Fats | i. 10-15% of total energy |
| 1. Proteins | ii. 3-5 grams |
| 1. Carbohydrates | iii. 20-30% of total energy |
| 1. Salt | iv. 50-60% of total energy |

- 1. A-iii, B-iv, C-i, D-ii
  2. A-iii, B-i, C-iv, D-ii
  3. A-i, B-iii, C-iv, D-ii
  4. A-iv, B-i, C-iii, D-ii

1. **Match the following. (1 Mark)**

| **Colum A** | **Colum B** |
| --- | --- |
| 1. Smokeless tobacco | i. Smoking by person sitting next to you |
| 1. Smoking | ii. Cigarette |
| 1. Most common cancer in males in India | iii. Gutkha |
| 1. Most common cancer in females in India | iv. Oral |
| 1. Secondhand smoking | v. Breast |

- 1. A-i, B-iii, C-iv, D-v, E-ii
  2. A-ii, B-i, C-v, D-iv, E-iii
  3. A-iii, B-ii, C-iv, D-v, E-i
  4. A-iii, B-i, C-v, D-iv, E-ii

1. **Match the columns for cancer & its danger sign- (1 Mark)**

| **Column A** | **Column B** |
| --- | --- |
| 1. Breast cancer | i. Blood in sputum |
| 1. Lung cancer | ii. Blood in stools |
| 1. Colon cancer | iii. Bad smell from mouth |
| 1. Oral cancer | iv. Mass in breast |

- 1. A-iii, B-ii, C-i, D-iv
  2. A-ii, B-i, C-iii, D- iv
  3. A-iv, B-i, C-ii, D-iii
  4. A-iv, B-iii, C-i, D-ii

**Mark True or False in given space for following questions (Q8-Q11):**

1. **Which of the following statements about cancers are true or false. (5 Marks)**

| **S.No.** | **Statement** | **True/False** |
| --- | --- | --- |
| **1** | Cancer is infectious and can spread from one person to another. |  |
| **2** | 1/3^rd^ of all cancer can be cured if they are diagnosed in early stages. |  |
| **3** | Cancer is only a familial disease. |  |
| **4** | Operating on a cancer will make it spread to other parts in body. |  |
| **5** | Cancer can only be treated with extensively tested drugs, radiation, or surgery. |  |

1. **Presence of following is a dander sign of cancer – (5 Marks)**

| **S.No.** | **Statement** | **True/False** |
| --- | --- | --- |
| **1** | A lump or hard area in the breast. |  |
| **2** | A change in a wart or mole |  |
| **3** | Evening rise of temperature |  |
| **4** | Unexplained loss of weight |  |
| **5** | A persistent change in digestive and bowel habit |  |

1. **Which of the following statements are true about healthy diet - (5 Marks)**

| **S.No.** | **Statement** | **True/False** |
| --- | --- | --- |
| **1** | 3 cups of food from dairy group provides required amount of calcium in a day |  |
| **2** | Calcium is found in only milk products |  |
| **3** | Half of the grains in daily food should be whole grains |  |
| **4** | Fruits and vegetables should be at least half of the food plate |  |
| **5** | Fruit juices are better than fruits |  |

1. **State whether following statement is true or false – (5 Marks)**

| **S.No.** | **Statement** | **True/False** |
| --- | --- | --- |
| **1** | Palliative care can only be given at hospital. |  |
| **2** | Palliative care is also known as supportive care. |  |
| **3** | The goal of palliative care is to cure the patient of disease |  |
| **4** | The goal of palliative care is to prevent or treat the symptoms and side effects of the disease and its treatment. |  |
| **5** | Besides physical care, palliative care also includes emotional and spiritual component. |  |
