# Supplementary material for: Improving adolescents’ dietary behavior through teacher-delivered cancer prevention education: a school-based cluster randomized intervention trial in urban Rajasthan
Source: BMC Public Health. 2024 Feb 28;24:630. doi: 10.1186/s12889-024-18114-8 (PMC10900637; doi:10.1186/s12889-024-18114-8)
Supplement: Supplementary file 2 — Supplementary Material 2 [file 12889_2024_18114_MOESM2_ESM.docx]

**Annexure 2**

| **Table:** Effect of intervention on teachers' knowledge towards cancers | | | | |
| --- | --- | --- | --- | --- |
| ***Variable*** | ***Pre-test***  *Mean (SD)* | ***Post-test***  *Mean (SD)* | ***Mean difference***  ***(95% CI)*** | ***P value*** |
| 1. Knowledge regarding cancer (Max score = 19) | 13.63  (1.71) | 18.56  (0.51) | 4.93  (4.02, 5.86) | <0.001^*^ |
| a. Biology of cancer  (Maximum score = 7) | 5.63  (1.26) | 6.94  (0.25) | 1.31  (0.62, 2.01) | 0.001^*^ |
| b. Risk factors and danger  signs (Maximum score = 7) | 4.94  (0.44) | 6.69  (0.48) | 1.75  (1.39, 2.11) | <0.001^*^ |
| c. Palliative care  (Maximum score = 5) | 3.06  (0.57) | 4.94  (0.25) | 1.88  (1.55, 2.21) | <0.001^*^ |
| 2. Diet and health  (Maximum score = 8) | 5.13  (1.15) | 7.50  (0.63) | 2.37  (1.68, 3.07) | <0.001^*^ |
| Total test score ^†^  (Maximum score = 27) | 18.76  (2.30) | 26.06  (0.57) | 7.30  (6.20, 8.43) | <0.001^*^ |
| *Paired t-test is used for analyzing significance of change in mean scores.*  *^*^ Statistically significant at confidence level of 95%*  ^†^ *Total test score is sum of 1 + 2* | | | | |
