# Supplementary material for: Improving adolescents’ dietary behavior through teacher-delivered cancer prevention education: a school-based cluster randomized intervention trial in urban Rajasthan
Source: BMC Public Health. 2024 Feb 28;24:630. doi: 10.1186/s12889-024-18114-8 (PMC10900637; doi:10.1186/s12889-024-18114-8)
Supplement: Supplementary file 3 — Supplementary Material 3 [file 12889_2024_18114_MOESM3_ESM.docx]

**Annexure 3**

| **Table:** Baseline scores for TPB constructs towards dietary behaviors | | | |
| --- | --- | --- | --- |
| ***TPB Measures*** | ***Baseline scores for TPB construct,*** *Mean (SD)* | | ***P value*** |
|  | ***Intervention group***  *(n=530)* | ***Non-intervention group*** *(n=566)* |  |
| ***Limiting consumption of fried/fast/packed food & sugar sweetened beverages*** | | | |
| Attitude | 6.40 (2.25) | 5.83 (2.45) | <0.001*^*^* |
| Subjective norms | 7.40 (1.81) | 7.05 (2.02) | 0.003*^*^* |
| Perceived behavioral control | 8.45 (1.80) | 8.46 (1.96) | 0.930 |
| Intention to limit consumption | 4.05 (1.25) | 4.06 (1.36) | 0.899 |
| ***Consuming fruits & vegetables daily*** | | | |
| Attitude | 8.88 (1.17) | 9.16 (1.13) | <0.001*^*^* |
| Subjective norms | 8.67 (1.55) | 8.67 (1.57) | 1.000 |
| Perceived behavioral control | 8.81 (1.62) | 8.82 (1.64) | 0.916 |
| Intention to consume daily | 4.52 (0.91) | 4.53 (0.96) | 0.860 |
| *-Composite scores for Attitude, SN, and PBC were on a scale of 2 to 10 and interpreted as follows : 8 and above scores – Good, while 2 to 7 scores as poor. For intention, the scores were scaled from 1 to 5 points with scores of 4 and more – Good, while 1 to 3 as poor.*  *^*^ Statistically significant at confidence level of 95%* | | | |
