# Supplementary material for: Improving adolescents’ dietary behavior through teacher-delivered cancer prevention education: a school-based cluster randomized intervention trial in urban Rajasthan
Source: BMC Public Health. 2024 Feb 28;24:630. doi: 10.1186/s12889-024-18114-8 (PMC10900637; doi:10.1186/s12889-024-18114-8)
Supplement: Supplementary file 4 — Supplementary Material 4 [file 12889_2024_18114_MOESM4_ESM.docx]

| ***Table:*** Comparison of baseline dietary behaviors in intervention and nonintervention groups | | | |
| --- | --- | --- | --- |
| ***Variable*** | ***Intervention group*** *(n=530)* | ***Nonintervention group*** *(n=566)* | ***P value^a^*** |
| Limiting fried/fast/packed food & Sugar Sweetened Beverages | 260 (49.1%) | 269 (47.5%) | 0.612 |
| Limiting at least one of – fried/fast food, and packed food & SSB | 407 (76.8%) | 406 (71.7%) | 0.056 |
| Consuming fruits & vegetables daily | 316 (59.6%) | 355 (62.7%) | 0.293 |
| Consuming at least one of fruits & vegetables daily | 486 (91.7%) | 518 (91.5%) | 0.915 |
| ^a^ *Chi-square test is used* | | | |

**Annexure 4**
